# Recombinant human adenovirus-p53 therapy for the treatment of nasopharyngeal carcinoma: a meta-analysis

**DOI:** 10.1186/s40064-016-3574-6

**Published:** 2016-10-27

**Authors:** Cheng Yuan, Xin-Hua Xu, Zhuo Chen

**Affiliations:** 1 The First College of Clinical Medical Science, China Three Gorges University & Yichang Central People’s Hospital, Yichang, 443003 Hu Bei China; 2 Department of Oncology, China Three Gorges University & Yichang Central People’s Hospital, Yi Ling Road 183, Yichang, 443003 Hu Bei China

**Keywords:** Nasopharyngeal carcinoma, Recombinant human adenovirus-p53, Chemoradiotherapy, Radiotherapy, Meta-analysis

## Abstract

To compare clinical curative effects and toxicity of recombinant human adenovirus-p53 injection (rAd-p53, Gendicine) combining chemoradiotherapy (CRT)/radiotherapy (RT) with those obtained with CRT/RT alone in nasopharyngeal carcinoma (NPC). We searched all the eligible studies from the Pubmed, Cochran Library, Embase, Web of science, Wanfang database and Chinese National Knowledge Infrastructure (CNKI). A total of twelve studies including 566 participants met the criteria to perform a meta-analysis. The results indicated the complete remission (CR) and overall response (OR) in the combination therapy group were significantly improved compared with the CRT/RT group (CR:RR = 2.03, 95% CI 1.66–2.48, p < 0.00001; OR:RR = 1.23, 95% CI 1.13–1.33, p < 0.00001), and patients who received the combination therapy showed significantly prolonged 1- and 2-year overall survival (OS), 2 year disease-free survival (DFS) rate and 2 year recurrence-free survival (RFS) rate (1 year OS:RR = 1.08, 95% CI 1.00–1.17, p = 0.04; 2 year OS:RR = 1.12, 95% CI 1.00–1.26, p = 0.04; 2 year DFS:RR = 1.41, 95% CI 1.09–1.83, p = 0.008; 2 year RFS:RR = 1.16, 95% CI 1.03–1.31, p = 0.02), but there was no significance in 3 year OS rate and 2 year distant metastases-free survival (DMFS) rate (3 year OS:RR = 1.28, 95% CI 1.00–1.62, p = 0.05; 2 year DMFS:RR = 1.05, 95% CI 0.89–1.24, p = 0.55). Furthermore, CRT/RT combined with rAd-p53 could not aggravate the myelosuppression versus CRT/RT alone (RR = 0.79, 95% CI 0.51–1.23, p = 0.30). The results demonstrated CRT/RT combined with rAd-p53 can result in enhanced survival and better clinical responses of patients with NPC. Therefore, rAd-p53 has great potential as an effective therapy for NPC.

## Background

Nasopharyngeal carcinoma (NPC), arising from the nasopharynx epithelium, is a kind of head and neck cancers (Chua et al. 2015). Worldwide, 86,500 cases were diagnosed with NPC in 2012, and the most of new cases were in China, Southeast Asia and other Asian countries. In recent years, Concurrent chemoradiotherapy (CRT) is being used in the treatment of NPC, gradually becoming the predominant method for the treatment of advanced NPC. However, the 5 year survival rate is only 40–50%.

As we know, the p53 tumor suppressor gene mutations or deletions are associated with malignant transformation and tumor progression (Lo et al. 2004; Golubovskaya et al. 2009; Olivier et al. 2002). The wildtype p53-induced phosphatase 1 (Wip1) protein was increased in advanced NPC, which may involve in the processes of proliferation, apoptosis, migration and invasion (Sun et al. 2015). Recombinant human adenovirus-p53 injection (rAd-p53, Gendicine) has been used for the clinical treatment of NPC. Despite the potential advantages of rAd-p53 for NPC, it is still not clear whether CRT/RT combined with rAd-p53 might be better than CRT/RT alone. Here, we performed a meta-analysis on twelve published clinical randomized controlled trials (RCTs) to derive a more precise evaluation of the effect of rAd-p53 in the treatment NPC.

## Methods

### Search strategy

A systematic literature search was performed though Pubmed, Cochran Library, Embase, Web of science, Wanfang database, Chinese National Knowledge Infrastructure (CNKI), covering all articles published up to October 2015. The search terms included “Recombinant human adenovirus-p53”, “rAd-p53”, “Gendicine”, “nasopharyngeal carcinoma” and “nasopharyngeal neoplasms”. The language was English or Chinese. When overlapping articles were found, we only included the publications that reported the most extensive information.

### Selection criteria

This meta-analysis aimed to evaluate the impact of combination therapy on patient survival, clinical responses and safety. So the selection criteria for this study were as follows: Firstly, trials were eligible for the present meta-analysis if they were randomized controlled trials (RCTs) of patients with NPC. Secondly, the patients in the experimental group received CRT/RT combined with rAd-p53, whereas patients in the control group were treated using CRT/RT alone. Thirdly, the endpoints were patient survival, clinical responses and safety.

### Data extraction and quality assessment

The extraction of data was operated independently by two investigators (Cheng Yuan and Zhuo Chen). Disagreements were resolved through discussion with a third investigator (Xin-hua Xu). Then the following relevant information was extracted from the selected studies: the first author’s surname, year of publication, number of patients, tumor stages, CRT/RT regimens and dose of RAd-p53.

The quality was evaluated by the Jadad composite scale (Jadad et al. 1996). This scale included the method of randomization (0–2 points), double blinding (0–2 points), and the description of dropouts (0–1 point).

### Curative effect evaluation

Survival outcomes were assessed in terms of the overall survival (OS), disease-free survival (DFS), recurrence-free survival (RFS) and distant metastases-free survival (DMFS) to evaluate prognosis, and treatment efficacy was assessed in terms of the overall response (OR) and complete response (CR). Toxicity event was assessed in terms of myelosuppression.

### Statistical analysis

Results were reported as pooled as relative risk (RR) and their 95% confidence interval (CI). Firstly, heterogeneity was identified. If the heterogeneity was not significant (p > 0.1, I^2^ < 50.0%), then the fixed-effect model can be performed, otherwise, the random effects model. Results of this meta-analysis were presented by forest plots, and the p value less than 0.05 was considered significant. Publication bias was evaluated though funnel plots. All analyses were performed by Review Manager (version 5.3, the Cochrane collaboration), using two side p values.

## Results

### Search results and characteristics of studies

Our search strategy identified 115 potentially relevant studies, and a total of twelve literatures were adopted in the end. The characteristics of the twelve studies (Zhang et al. 2006, 2007, 2011; Pan et al. 2009; Lan et al. 2011, 2012; Qin et al. 2012; Wang et al. 2010, 2012; Li 2012; Si et al. 2009; Chen et al. 2003) were summarized in Table 1. A total of 566 patients were included in the meta-analysis, of whom 298 received CRT/RT combined with rAd-p53 and 268 were treated with CRT/RT alone.

**Table 1:** Main characteristics of all the included studies

Studies	RAd-p53	RT	CT	Stages	Jadad
Qin et al. (2012)	1 × 1012 VP 1 time/week/6–7 weeks	Nasopharynx: 70–76 Gy/35–38f Neck: 66–70 Gy	Carboplatin: 500 mg/m^2^ Fluorouracil: 2000 mg/m^2^	III–IV	2
Lan et al. (2011)	1 × 1012 VP 1 time/week/6–8 weeks	Nasopharynx: 70–76 Gy/35f Neck: 60–70 Gy	Carboplatin: 500 mg/m^2^ 5-Fluorouracil: 2000 mg/m^2^	III–IV	2
Wang et al. (2010)	1 × 1012 VP Saline dilution 2–4 ml	Nasopharynx: 70 Gy/35f Neck: 70 Gy/35f	–	III–IV	2
Li (2012)	1 × 1012 VP –	Nasopharynx: 70–76 Gy/7–8 weeks Neck: 60–70 Gy	Carboplatin: 300–500 mg/m^2^ 5-Fluorouracil: 300–500 mg/m^2^	III–IV	2
Chen et al. (2003)	1 × 1012 VP 1 time/week/8 weeks	Nasopharynx: 70–76 Gy/7–8 weeks Neck: 60–70 Gy/7–8 weeks	–	II–IV	2
Si et al. (2009)	1 × 1012 VP 1 time/week/6–8 weeks	Nasopharynx: 70–76 Gy/7–8 weeks Neck: 36 Gy/4 weeks 60–70 Gy/3–4 weeks	Carboplatin: 300–500 mg/m^2^ 5-Fluorouracil: 300–500 mg/m^2^	III–IV	3
Wang et al. (2012)	1 × 1012 VP 1 time/week/9–10 weeks	Nasopharynx: 66–70 Gy/35f Neck: 66–70 Gy/35f	–	I–III	2
Zhang et al. (2006)	1 × 1012 VP 1 time/week/8 weeks	Nasopharynx: 70 Gy/35f Neck: 70 Gy/35f	–	Unclear	3
Zhang et al. (2011)	2 × 1012 VP 1 time/week/6 weeks	68–76 Gy to 60–70 Gy/6 weeks	Cisplatin: 75 mg/m^2^ Paclitaxel: 75 mg/m^2^	III–IV	3
Pan et al. (2009)	1 × 1012 VP 1time/week/8 weeks	Nasopharynx: 70 Gy/35f Neck: 70 Gy/35f	–	II–IV	3
Lan et al. (2012)	1 × 1012 VP 1 time/3 days/6–8 times	70–76 Gy/35–38f	Cisplatin: 300 mg/m^2^ 5-Fluorouracil: 2000 mg/m^2^	Unclear	2
Zhang et al. (2007)	1 × 1012 VP 1 time/1 week/8 times	Nasopharynx: 70 Gy/35f Neck: 70 Gy/35f	–	Unclear	3

*RAd*-*p53* recombinant human adenovirus-p53 injection; *RT* radiotherapy; *CT* chemotherapy

### Recent efficacy assessments

**Fig. 1:**
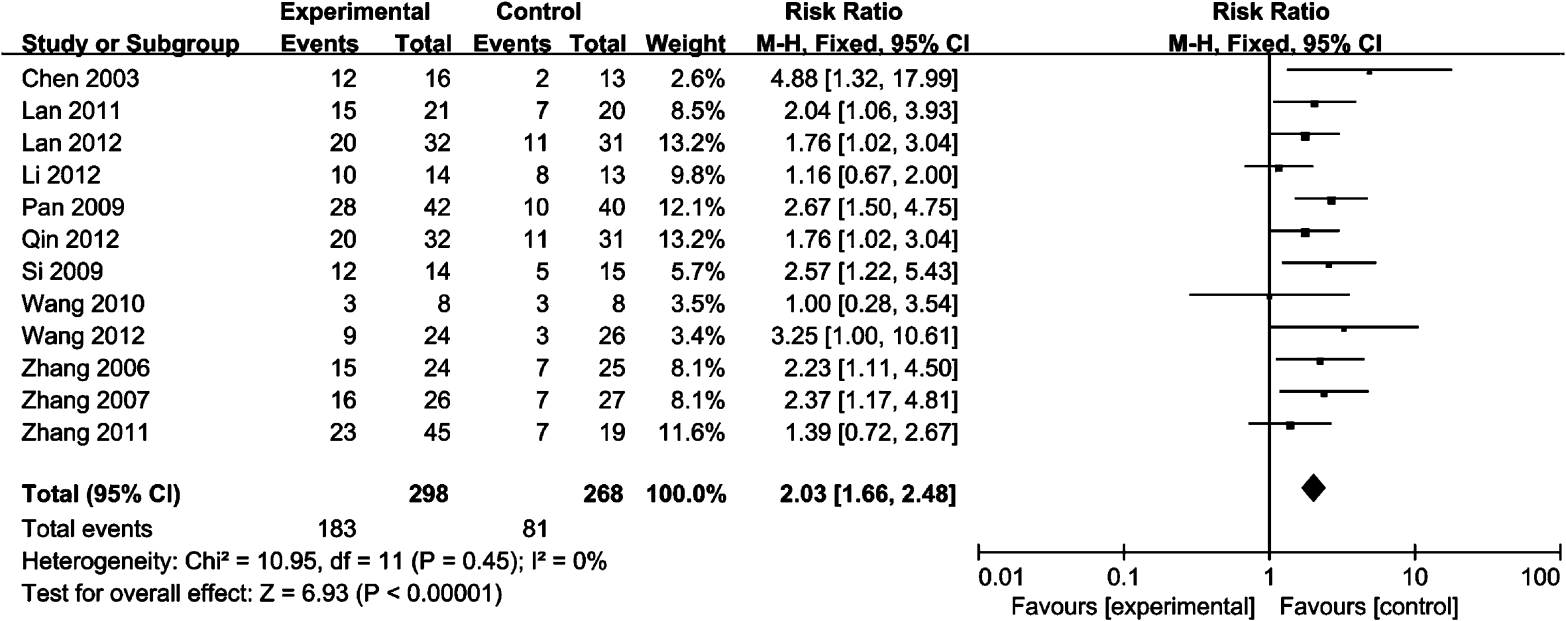
Forest plot for the meta-analysis of complete remission (CR) rate of primary tumor

**Fig. 2:**
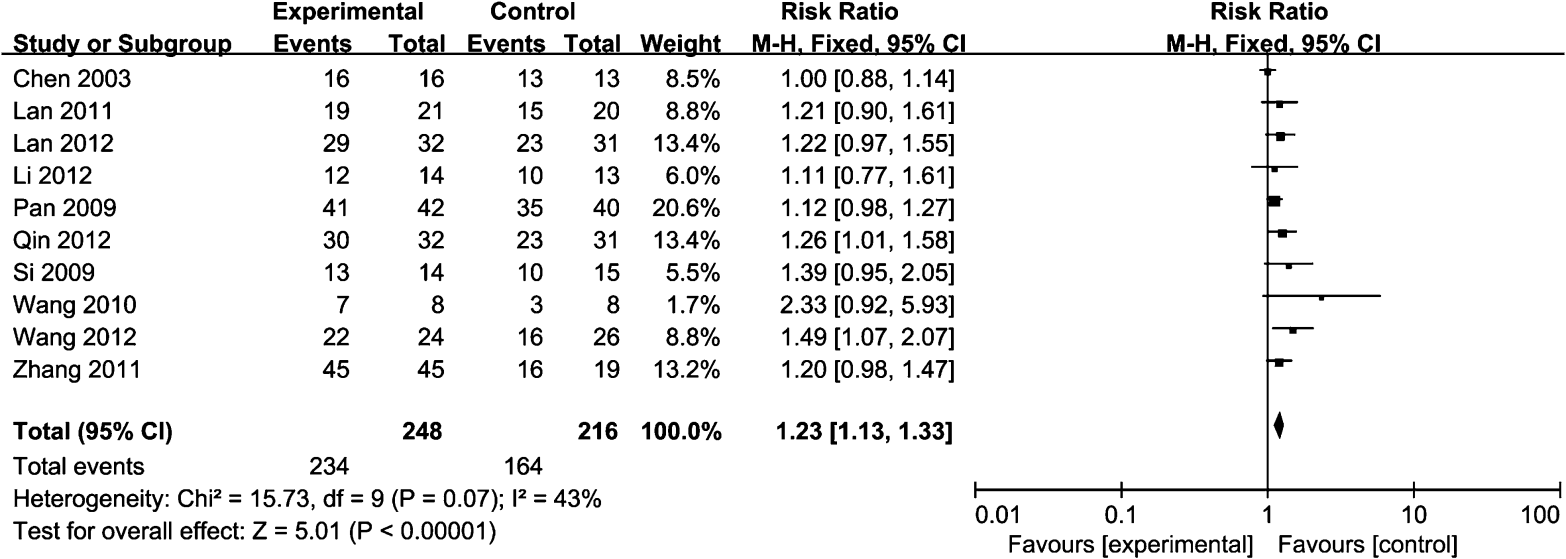
Forest plot for the meta-analysis of overall response (OR) rates of primary tumor

Recent efficacy was assessed in terms of CR and OR. Twelve studies compared CR, of which 298 patients were treated with CRT/RT combined with rAd-p53 and 268 patients were treated with CRT/RT alone, and ten trials including 464 patients compared OR. As shown in Figs. 1, 2, the analysis results indicated that the CR and OR in the combination therapy group were significantly improved compared with the CRT/RT group (CR:RR = 2.03, 95% CI 1.66–2.48, p < 0.00001; OR:RR = 1.23, 95% CI 1.13–1.33, p < 0.00001).

### Survival outcome

**Fig. 3:**
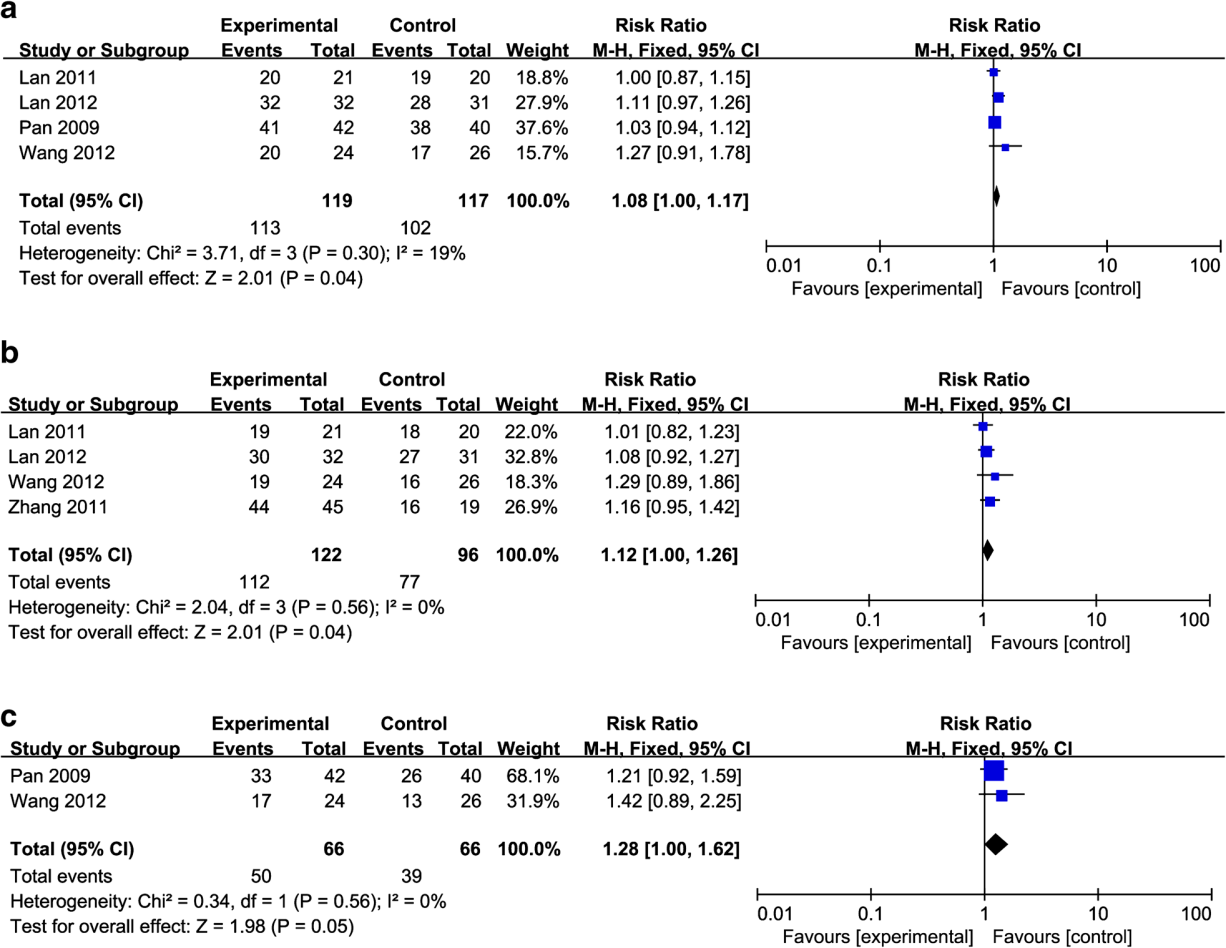
Forest plot for the meta-analysis of the 1-, 2- and 3-year overall survival (OS) rate

There were four eligible studies that reported the endpoints of 1- and 2-year OS rate. CRT/RT combined with rAd-p53 showed significantly increased 1- and 2-year OS rates compared with the rates observed when CRT/RT alone was used (1-year OS:RR = 1.08, 95% CI 1.00–1.17, p = 0.04, Fig. 3a and 2-year OS:RR = 1.12, 95% CI 1.00–1.26, p = 0.04, Fig. 3b). In addition, two studies compared 3 year OS, and the result of meta-analysis showed there was no significance in the outcome of 3-year OS versus control (RR = 1.28, 95% CI 1.00–1.62, p = 0.05; Fig. 3c).

**Fig. 4:**
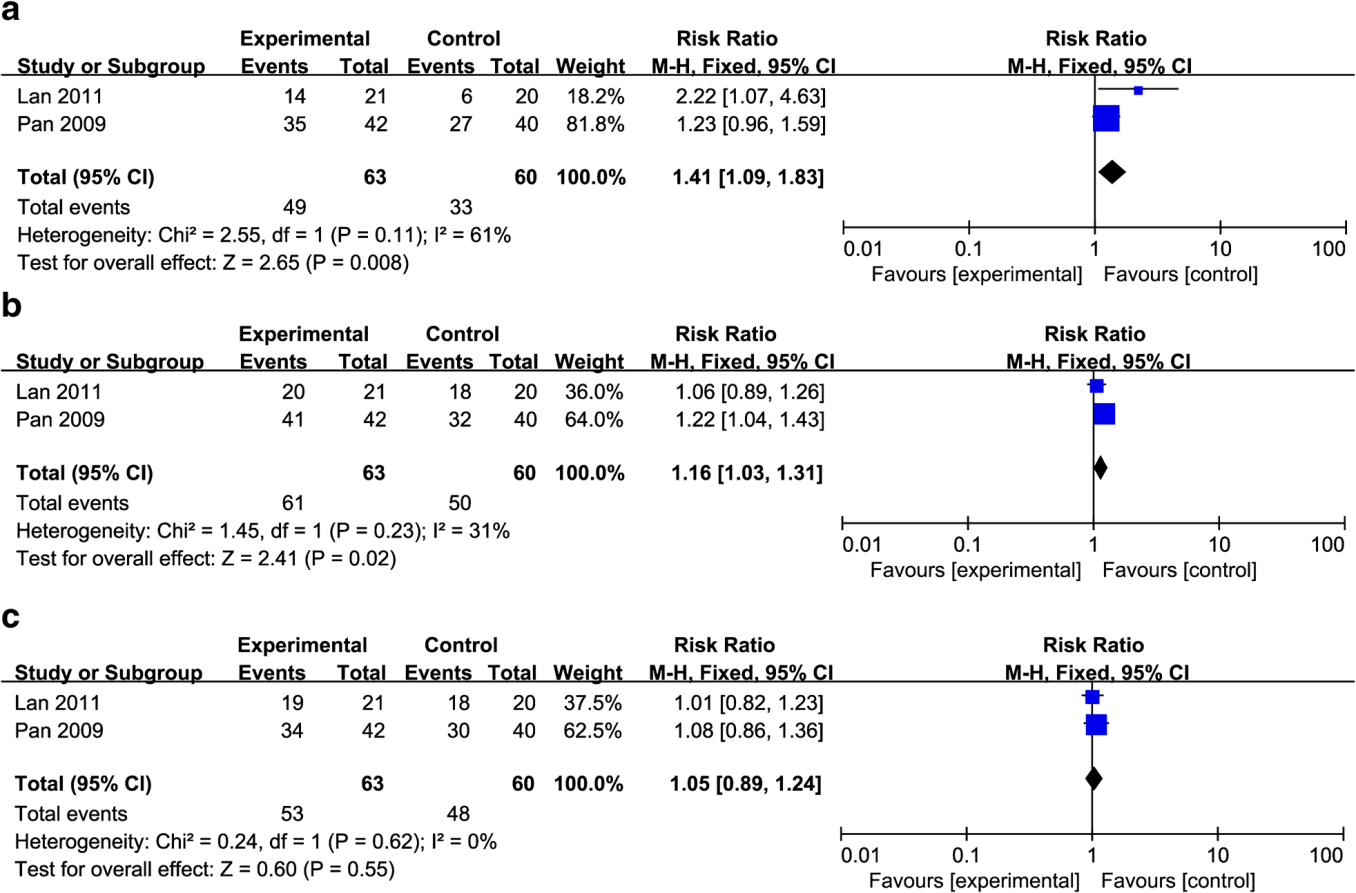
Forest plot for the meta-analysis of the 2 year disease-free survival (DFS) rate, recurrence-free survival (RFS) rate and distant metastases-free survival (DMFS) rate

As shown in Fig. 4, patients who received the combination therapy showed significantly prolonged 2 year DFS rate and 2 year RFS rate compared with patients who received CRT/RT alone, but there was no significance in 2 year DMFS rate (2 year DFS:RR = 1.41, 95% CI 1.09–1.83, p = 0.008, Fig. 4a; 2-year RFS:RR = 1.16, 95% CI 1.03–1.31, p = 0.02, Fig. 4b; 2-year DMFS:RR = 1.05, 95% CI 0.89–1.24, p = 0.55, Fig. 4c).

### Toxicity

**Fig. 5:**
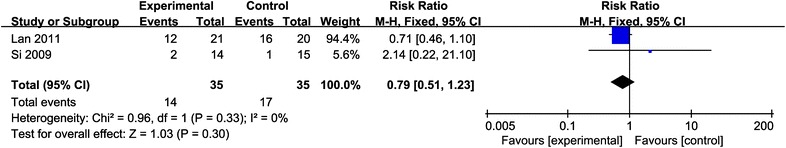
Forest plot for the meta-analysis of the occurrence of myelosuppression

Data available regarding the toxicities were limited. The reporting and occurrence of adverse events was rare. The most common side effect was fever which was reported, but it was not compared between the combination therapy group and CRT/RT group. Only 2 of the 12 trials reported the occurrence of myelosuppression. The result indicated CRT/RT combined with rAd-p53 could not aggravate the myelosuppression compared with CRT/RT alone (RR = 0.79, 95% CI 0.51–1.23, p = 0.30; Fig. 5).

### Heterogeneity and publication bias

**Fig. 6:**
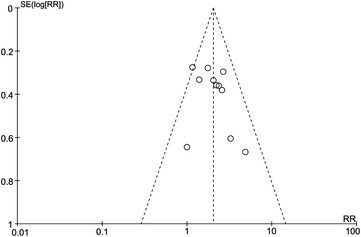
The funnel plot of publication bias

There was no evidence of heterogeneity in all the recent efficacy, survival outcomes and toxicity in the Chi square and I-square tests, and a fixed effect model was used. Funnel plot was performed to assess the publication bias in all the included studies for evaluation of the CR. Since no more than 10 studies were included in the OR, survival outcomes and toxicity, funnel plot was not performed. As shown in Fig. 6, the funnel plot did not reveal any evidence of significant asymmetry in CR.

## Discussion

To our knowledge, this is the first meta-analysis to explore the curative efficacy of rAd-p53 in the treatment of NPC. During the past decades, despite progress in technique evolution of RT, the long-term survival rate of NPC did not receive fundamental improvement, so that developing new effective therapeutic modality are urgent need (Mitchell et al. 2010). In 2003, a gene therapy product (non-replicating adenovirus) received approval for the treatment of head and neck cancer, and then a replication-selective adenovirus was used to treat NPC (Hughes et al. 2012). Nevertheless, it is very necessary to have a systematic comparison for the curative effects and toxicity between CRT/RT combined with rAd-p53 and CRT/RT alone.

In our study, our meta-analysis including data from twelve published studies with 566 patients indicated that CR and OR in the combination therapy group were significantly improved compared with the CRT/RT group. Combination therapy showed significantly prolonged 1- and 2-year OS rate, 2 year DFS rate and 2 year RFS rate. While, there was no significance in 3 year OS rate and 2 year DMFS rate. In addition, CRT/RT combined with rAd-p53 could not aggravate the myelosuppression versus CRT/RT alone.

For this reason that adeno-associated virus (AAV) cannot replicate on its own, recombinant adeno-associated viruses (rAAV) garnered much attention in the field of gene therapy to the cancer, which can overcome transduction barriers at the level of receptor binding, subcellular trafficking, and transgene expression (Mitchell et al. 2010; Nicolson and Samulski 2014). The wild-type p53 gene (wt-p53) influences cell survival and the response to radiation-induced DNA damage. The studies (Hirao et al. 2000; Ma et al. 2012) found activated p53 transcription promotes can lead to cell cycle arrest, DNA repair, tumor cell apoptosis, and even killing effect on tumor cells. A previous study (Kandioler et al. 2002) suggested that patients were less sensitive to RT if the function of wt-p53 lost, and RT combined with rAd-p53 may increase the sensitivity in vivo and in vitro studies (Shiomitsu et al. 2008; Gudkov and Komarova 2003; Cuddihy and Bristow 2004). RAd-p53 can carry the recombinant human p53 gene and then introduce the p53 gene into tumor cells to express wt-p53 protein, inhibiting cell division and inducing tumor cell apoptosis (Pan et al. 2009).

However, the limitations of the study cannot be ignored. Firstly, the twelve trials included in the meta-analysis were mainly concentrated in the Chinese population, and this may be related to the high incidence of NPC in China. Secondly, there was limited information regarding some patients, and the total sample sizes were small. Thirdly, literatures were searched by English and Chinese, which may lead to potential publication bias, although publication bias was not significant in the study.

In conclusion, our results demonstrated that CRT/RT combined with rAd-p53 therapy can result in enhanced survival and better clinical responses of patients with NPC. Therefore, rAd-p53 has great potential as an effective therapy for NPC.

## Abbreviations

CRT: chemoradiotherapy
RT: radiotherapy
rAd-p53: recombinant human adenovirus-p53
NPC: nasopharyngeal carcinoma
RR: relative risk
CI: confidence interval
CR: complete remission
OR: overall response
OS: overall survival
Wip1: wildtype p53-induced phosphatase 1
RCT: randomized controlled trial
DFS: disease-free survival
RFS: recurrence-free survival
DMFS: distant metastases-free survival
